# EZH2 is a potential prognostic predictor of glioma

**DOI:** 10.1111/jcmm.16149

**Published:** 2020-12-04

**Authors:** Yi‐nan Chen, Shi‐qiang Hou, Rui Jiang, Jun‐long Sun, Chuan‐dong Cheng, Zhong‐run Qian

**Affiliations:** ^1^ Department of Neurosurgery The First Affiliated Hospital of University of Science and Technology of China Division of Life Sciences and Medicine Hefei China; ^2^ Department of Neurosurgery Chuzhou Clinical College of Anhui Medical University The First People's Hospital Chuzhou Chuzhou China; ^3^ Jiangsu Clinical Medicine Center of Tissue Engineering and Nerve Injury Repair and Department of Neurosurgery Affiliated Hospital of Nantong University Nantong China; ^4^ Department of Neurosurgery Shanghai Jiao Tong University School of Medicine Affiliated Renji Hosipital Shanghai China

**Keywords:** EZH2, glioma, immunity, overall survival

## Abstract

The enhancer of zeste homologue 2 (EZH2) is a histone H3 lysine 27 methyltransferase that promotes tumorigenesis in a variety of human malignancies by altering the expression of tumour suppressor genes. To evaluate the prognostic value of EZH2 in glioma, we analysed gene expression data and corresponding clinicopathological information from the Chinese Glioma Genome Atlas, the Cancer Genome Atlas and GTEx. Increased expression of EZH2 was significantly associated with clinicopathologic characteristics and overall survival as evaluated by univariate and multivariate Cox regression. Gene Set Enrichment Analysis revealed an association of EZH2 expression with the cell cycle, DNA replication, mismatch repair, p53 signalling and pyrimidine metabolism. We constructed a nomogram for prognosis prediction with EZH2, clinicopathologic variables and significantly correlated genes. EZH2 was demonstrated to be significantly associated with several immune checkpoints and tumour‐infiltrating lymphocytes. Furthermore, the ESTIMATE and Timer Database scores indicated correlation of EZH2 expression with a more immunosuppressive microenvironment for glioblastoma than for low grade glioma. Overall, our study demonstrates that expression of EZH2 is a potential prognostic molecular marker of poor survival in glioma and identifies signalling pathways and immune checkpoints regulated by EHZ2, suggesting a direction for future application of immune therapy in glioma.

## 1 INTRODUCTION

Gliomas are the most common and malignant type of primary intracranial tumour, representing about 28% of all primary tumours and approximately 80% of malignant tumours in the brain.^1^ The World Health Organization categorizes gliomas as histological grades I‐IV, which correspond to their degrees of malignancy.^2^ The most aggressive and common form, glioblastoma (GBM; about 45% of gliomas), has a 5‐year survival rate of only about 5%.^3^ Widespread infiltration of healthy brain tissue by glioma cells is largely responsible for poor prognosis and makes it difficult to find curative therapies.^4^ Combined surgery with focal fractionated radiotherapy and the adjuvant temozolomide has become the standard first line treatment for newly diagnosed GBM^5^; however, the median survival of GBM patients remains less than 15 months.^6^ Therefore, there is an urgent need to clarify potential mechanisms underlying the development of glioma in order to identify novel diagnostic biomarkers and potential therapeutic targets.

Immune checkpoints have been shown to play important roles in glioma immune escape. Immune therapies with immuno‐checkpoint blocking monoclonal antibodies, cell therapy, DNA vaccine or small molecule inhibitors have shown clinical efficacy in a number of tumour types, including glioma, melanoma, non‐small cell lung cancer, renal cancer and bladder cancer.^7^, ^8^, ^9^, ^10^, ^11^ Checkpoint inhibitors, including programmed death‐ligand 1 and programmed cell death protein 1 inhibitors, have emerged as potential therapeutic options for low grade glioma (LGG) and GBM.^12^ Furthermore, the tumour mutational burden (TMB) has been reported as an independent prognostic factor for glioma.^13^ Patients with a higher TMB exhibit shorter overall survival. Moreover, microsatellite instability (MSI) has been identified as an independent prognostic factor in several tumour types..^14^, ^15^, ^16^ Microsatellites are extremely prone to DNA replication errors that are readily corrected by DNA mismatch repair (MMR) system.^17^ Thus, increased understanding of immune checkpoint, TMB and MMR pathways could provide a context for understanding glioma disease progression.

Enhancer of zeste homologue 2 is a catalytic subunit of the polycomb repressive complex 2.^18^ It is a histone methyltransferase that tri‐methylates histone H3 at Lys 27 (H3K27me3) in mammalian cells.^19^ Accumulated evidence suggests that EZH2 is involved in tumorigenesis, affecting cell proliferation and apoptosis, epithelial to mesenchymal transition, invasion, and drug resistance in gliomas and other cancers.^20^, ^21^, ^22^, ^23^ Inhibition of EZH2 by small molecular inhibitors or gene knockdown results in reduced cancer cell growth and tumour formation.^19^ In addition, EZH2 expression in immune cells in the tumour microenvironment is reported to have a direct role in mediating the T cell reaction.^24^ In this study, we investigated the prognostic value of EZH2 in glioma. Moreover, we characterized signal pathways regulated by EZH2 by using gene set enrichment analysis (GSEA). To best understand the immune relevance of EZH2, we conducted correlation analysis of EZH2 in the tumour microenvironment and immune infiltration and generated a nomogram of pathways associated with EZH2 expression and prognosis. Our results provide comprehensive understanding of the molecular mechanism of EZH2 in glioma, with potential relevance to prognosis and the development of novel therapies.

## 2 MATERIALS AND METHODS

### 2.1 Cell culture

Human astrocytes (HA‐1800) and glioma cell lines (U87 and U251) were purchased from ATCC (the American Type Culture Collection). STR cell authentication was conducted by Cobioer Biosciences CO., LTD. All of the cells were cultured in high‐glucose DMEM containing 10% foetal bovine serum (FBS, Gibco) and incubated at 37°C in a humidified incubator with 5% CO_2_.

### 2.2 Quantitative real‐time PCR (qRT‐PCR)

Total RNA from cultured cells was extracted using Trizol reagent (Life Technologies Corporation). The first cDNA strand was synthesized using a RevertAid™ First Strand cDNA Synthesis kit (Fermentas). qRT‐PCR was performed on a Corbett RG‐6000 polymerase chain reaction system (Qiagen), using FastStart Universal SYBR‐Green Master Mix (Roche) to detect the expression of EZH2. β‐actin (Ruibo Bio) was used as an endogenous control. Fold changes were calculated using the relative quantification (2^−ΔΔCt^) method.

### 2.3 Western blotting

Cells were seeded into 6‐well plates at a density of 5 × 10^5^ cells/well for 48 hours. The cells were then washed in PBS and lysed in ice‐cold lysis buffer. The protein contents of the lysates were determined, and equal amounts of proteins were separated by sodium dodecyl sulphate‐polyacrylamide gel electrophoresis and then transferred onto polyvinylidene difluoride membranes (Bio‐Rad). Next, non‐specific interactions were blocked by incubation with 5% non‐fat milk‐tris‐buffered saline with 0.1% Tween‐20 at 37°C for 1 hour. The membranes were incubated with specific antibodies against EZH2 (1:4000; Proteintech) and β‐actin (1:2,000; Sigma) at 4°C for 12 hours to detect the corresponding proteins and then were washed and incubated with horseradish peroxidase‐conjugated secondary antibody (Bioss Antibodies) for 1 hour at 37°C. To analyse relative protein expression levels, immunoblots were detected with enhanced chemiluminescence reagents (Invitrogen Life Technologies) and exposed to X‐ray films. Quantity One software (Bio‐Rad) was used, with β‐actin as the internal reference.

### 2.4 Immunohistochemical staining

Tissue specimens from patients with different grades of glioma were fixed in formalin and embedded in paraffin for EZH2 immunohistochemistry (IHC) staining. After deparaffinization, hydration and blocking, the specimens were incubated with primary anti‐EZH2 antibody (Abcam ab186006; diluted 1:1000) overnight at 4°C. The sections were assessed microscopically by comparison of staining between each grade. The EZH2 expression was scored based on the intensity of staining and the percentage of cells at that intensity. The staining intensity was scored as 0 (no staining), 1 (weak intensity), 2 (moderate intensity) and 3 (strong intensity). The final staining scores were evaluated by two pathologists and calculated from the sum of the four intensity percentage scores. The outcome was analysed using SPSS 22.0.

### 2.5 Patient data and bioinformatics analysis

Normalized RNA‐seq and clinical data of glioma patients were downloaded from the CGGA website (http://www.cgga.org.cn/). Then, we identified the gene expression profiles and clinical data of 681 glioma cases for further analysis. The overall survival (OS) was considered the primary outcome. By utilizing the R programming language, we compared the standardized RNA‐seq data with the EZH2 gene expression data. The R ‘corrplot’ package was used to calculate the Pearson correlation at the transcriptional level. Box plots were used to visualize expression differences for discrete variables. We also identified the differential expression of EZH2 in various tissues and tumours by integrating the data sets from Genotype‐Tissue Expression (GTEx) and the Cancer Genome Atlas (TCGA).

### 2.6 Gene set enrichment analysis (GSEA)

Gene set enrichment analysis is a computational method used to identify hallmark gene sets with predicted statistically significant differences between two groups. In our study, we performed GSEA to elucidate the significant survival differences between groups with high and low EZH2 expression. Gene set permutations were performed 1000 times in each analysis to discover significant critical biological pathways. Pathways were considered significantly enriched if they had nominal *P* values <.05 and FDR <25%.

### 2.7 Independent prognostic factor evaluation

A nomogram‐based model was conducted to visualize the relationship between individual predictors and survival rates with the help of the R ‘rms’ package. We conducted univariate and multivariate cox regression analysis to evaluate whether our model can be used as an independent prognostic factor. Genes that were significantly correlated with EZH2 expression were also identified. The prognostic ability was evaluated by ROC and AUC analysis using the package of ‘survival ROC’ in R.

### 2.8 Immune correlation analysis

To investigate the relationship between tumour progression and immune response, we profiled the immune checkpoint expression in patients with LGG and GBM in data sets from TCGA. Furthermore, the amounts of tumour‐infiltrating lymphocytes (TILs) were also calculated. The analytical tool called CIBERSORT was used in the study, which can quantify the percentage of different types of TILs accurately, under the complex ‘gene signature matrix’. In the current study, we lustrated the immune infiltration of each sample with the preset signature matrix at 1000 permutations. After using the CIBERSORT program, the distribution of TILs was presented, along with the results of correlation coefficient, P‐value and root mean squared error (RMSE), which can evaluate the accuracy of the results in each sample. The Pearson correlation coefficient was used to calculate the association between the TMB and EZH2 expression. The relationship of MSI and EZH2 was calculated by the same procedure. To explore the composition of the tumour microenvironment, that ESTIMATE Score (Estimation of Stromal and Immune cells in Malignant Tumour tissues using Expression data) was calculated, and the presence of infiltrating stromal/immune cells in LGG or GBM tissues was evaluated. Using the ESTIMATE algorithm, we generated three scores: (a) a stromal score that captures the presence of stroma in tumour tissue, (b) an immune score that represents the infiltration of immune cells in tumour tissue and (c) an estimate score that infers tumour purity. The correlation between EZH2 expression and B cells, CD4 T cells, CD8 T cells, neutrophils, macrophages and dendritic cells was determined by using the TIMER database (https://cistrome.shinyapps.io/timer/).

### 2.9 Statistical analysis

All statistical data and figures were analysed by using SPSS 22.0 (IBM), R 3.3.1 (https://www.r‐project.org/) and GraphPad Prism 5.0. Correlations between two genes were analysed by the Pearson correlation method. The association between clinicopathologic characteristics and EZH2 was estimated with the Wilcoxon signed rank test and logistic regression. Kaplan‐Meier curves were generated, and log‐rank tests were performed to estimate the survival predictive performance of EZH2 and the risk score (RS). Univariate and multivariate Cox regression analyses were carried out to evaluate the relationship between variables and overall survival. The nomogram was created using the rms package of R software, and prognostic ability was evaluated using the package of ‘survival ROC’ in R. Statistical results with *P* < .05 were considered statistically significant.

## 3 RESULTS

### 3.1 EZH2 is overexpressed in glioma

**FIGURE 1:**
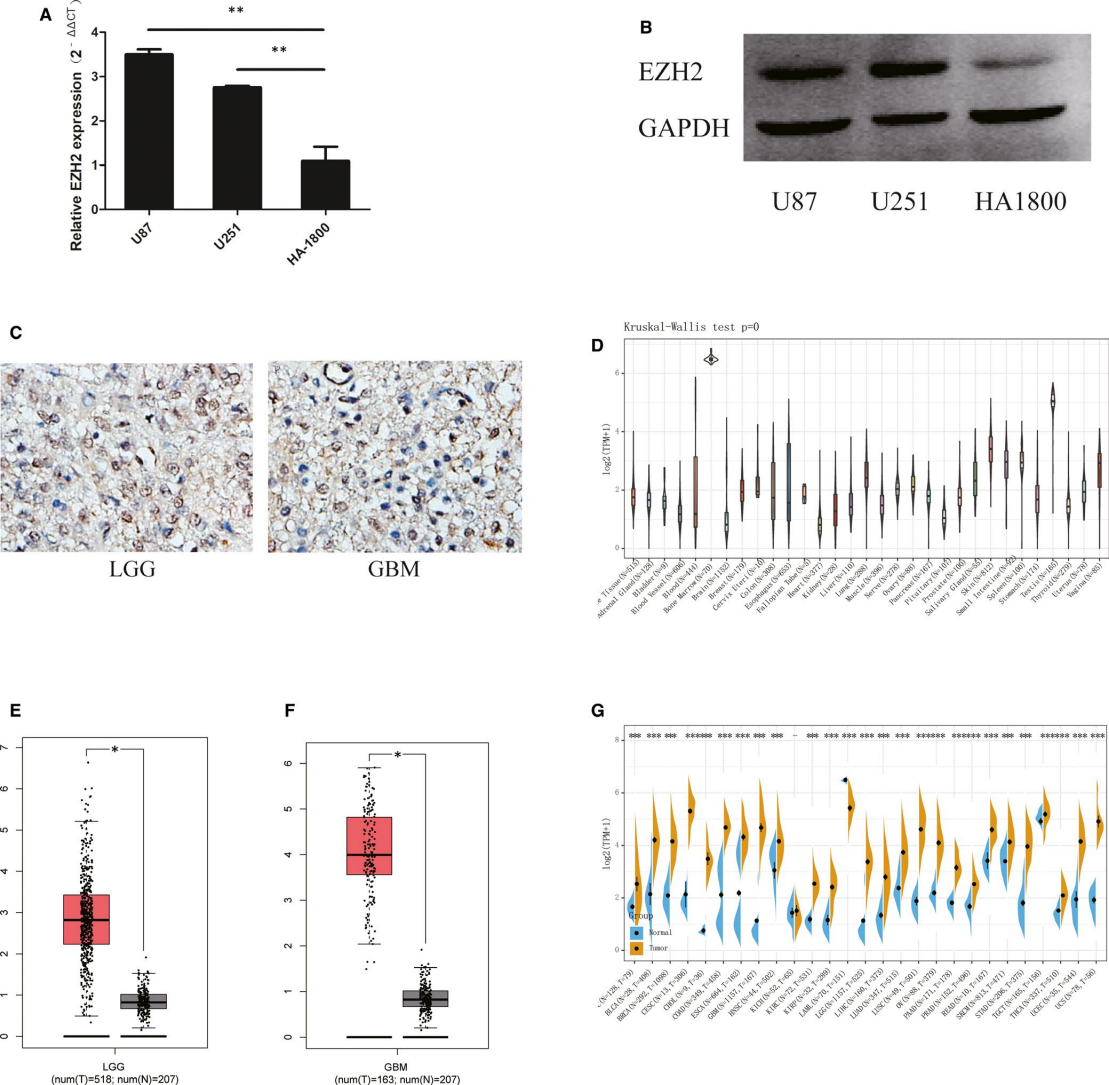
EHZ2 is overexpressed in glioma. (A) Relative qPCR expression levels of EZH2 in U87, U251 and HA‐1800 cells. (B) EZH2 protein expression levels in U87, U251 and HA‐1800 cells. (C) Representative IHC staining of EZH2 in low grade glioma (LGG) and glioblastoma (GBM) specimens. (D) The expression levels of EZH2 in normal tissues from the GTEx database. (E) Differential expression of EZH2 between LGG and normal tissues. (F) Differential expression of EZH2 between GBM and normal tissues. (G) The expression levels of EZH2 in various cancers from TCGA and GTEx databases. Data represent the results of three independent experiments. *P < .05 compared with the control

To examine the expression level of EZH2 in glioma cell lines, we performed qRT‐PCR and Western blotting. As shown in Figure 1A,B, EZH2 expression is significantly up‐regulated in the U87 and U251 glioma cells compared to HA‐1800 human astrocytes. IHC staining verified EZH2 staining in glioma tissues and showed that EZH2 expression is grade‐dependent, with higher expression levels in GBM than in LGG (Figure 1C and Figure S1). To further evaluate EZH2 expression patterns, we investigated the EZH2 levels in 518 LGG tissues, 163 GBM tissues and 207 normal tissues in the CGGA data set (Figure 1D). EZH2 expression was significantly increased in glioma tissues vs normal tissues, with more obvious difference for GBM tissues than for LGG tissues (Figure 1E,F). Furthermore, data from GTEx database verify that EZH2 expression is elevated in tumour from patients with glioma (Figure 1G).

### 3.2 EZH2 expression is associated with clinicopathologic variables and overall survival

**FIGURE 2:**
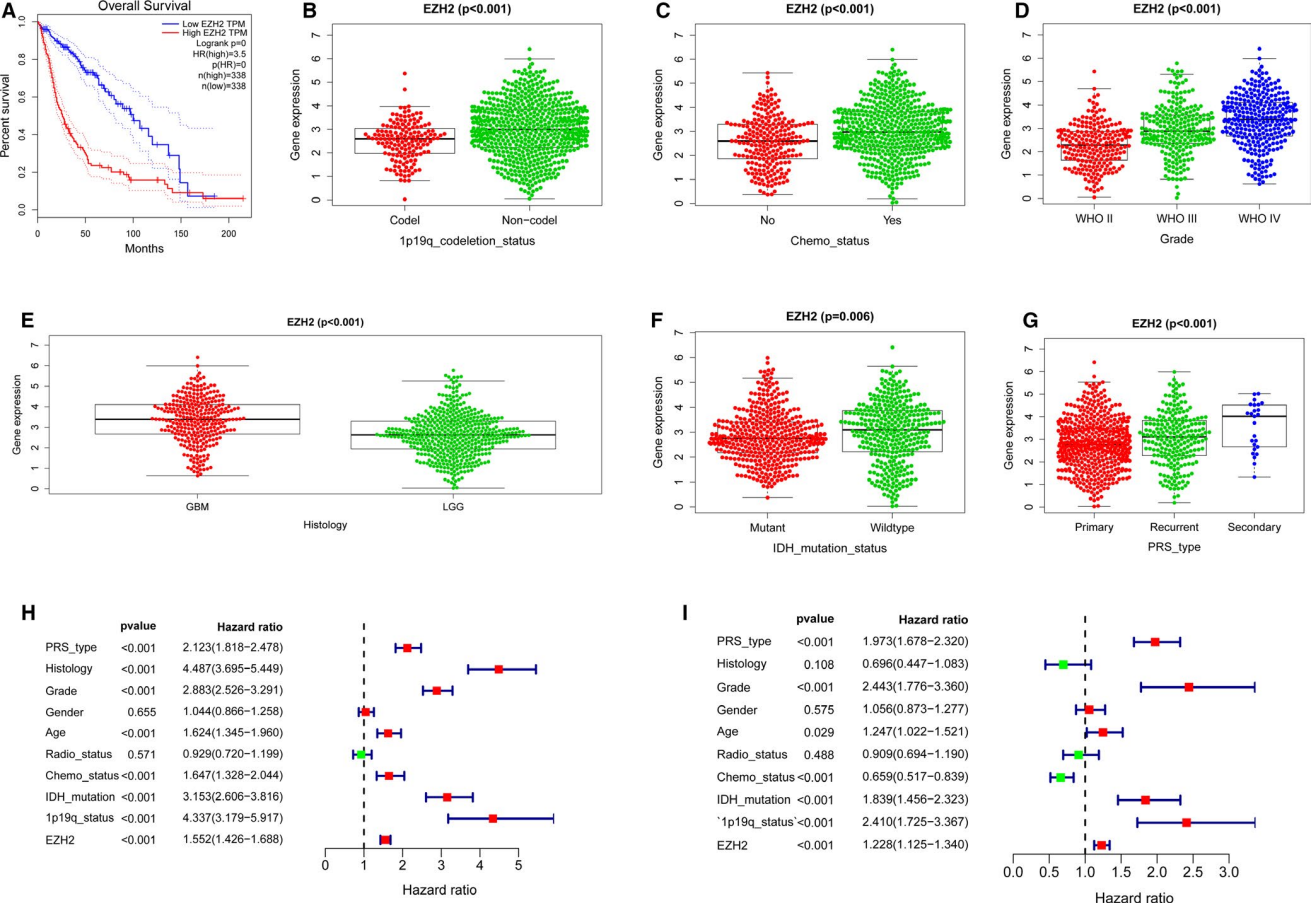
Relationship between EZH2 expression, clinicopathologic characteristics and overall survival. (A) Overall survival of glioma patients in high and low EZH2 expression groups from the CGGA database. (B‐G) Correlation of EZH2 expression with (B) 1p19q codeletion status, (C) Chemo status, (D) Grade, (E) Histology, (F) IDH mutation status and (G) PRS type. (H‐I) Forest plot showing univariate and multivariate cox regression analyses of EZH2 mRNA levels and clinicopathological variables predictive of overall survival

To verify previous results suggesting that EZH2 expression is associated with patient survival,^25^ we compared the overall survival (OS) of patients from the CGGA data set with low and high EZH2 expression (Figure 2A). Patients in the high‐EZH2 group had a shorter overall survival time than patients in the low‐EZH2 group, indicating that EZH2 expression may be associated with the survival of glioma patients.

To further evaluate the clinical significance of elevated EZH2 expression, we performed independent samples *t* tests. The results suggest that the expression of EZH2 correlates significantly with 1p19q codeletion status (*P* < .001; Figure 2B), Chemo status (*P* < .001; Figure 2C), grade (*P* < .001; Figure 2D), histology (*P* < .001; Figure 2E), PRS type (*P* < .001; Figure 2F) and IDH mutation status (*P* = .006; Figure 2G). Based on univariate Cox and multivariate Cox regression analysis, EZH2 expression was correlated with overall survival as an independent factor (Table S1). Univariate Cox analysis revealed that EZH2 expression, PRS type, Histology, Grade, Age, Chemo status, IDH mutation and 1p19q status were all associated significantly with the overall survival of glioma patients (Figure 2H). Furthermore, multivariate Cox regression analysis showed that high EZH2 expression correlated significantly with poor overall survival (HR = 1.228; *P* < .001). Other clinicopathologic parameters correlated with worse overall survival consisted of PRS type, Grade, Chemo status, IDH mutation and 1p19q status (Figure 2I). Therefore, our results indicate that EZH2 expression might serve as an independent prognostic factor of overall survival when adjusted by these variables.

### 3.3 Establishment of glioma prognostic prediction nomogram

**FIGURE 3:**
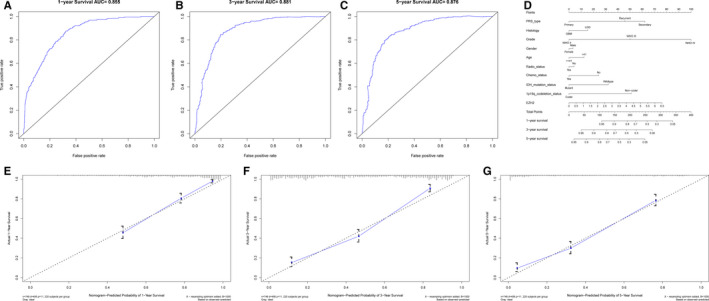
Evaluation of EZH2 expression as a prognostic indicator for glioma. (A‐C) ROC analysis of overall survival for EZH2 expression in the CGGA cohort at 1 y, 3 y or 5 y, respectively. (D) Nomogram to predict the overall survival of glioma patients based on clinical parameters and EZH2 expression. (E‐G): Nomogram‐predicted probabilities of 1‐, 3‐ and 5‐y survival

To further evaluate the prognostic ability of EZH2 expression in glioma, we performed receiver operating characteristic curve (ROC) analysis. The 1‐year survival area under the curve (AUC) of EZH2 expression was 0.855 (Figure 3A), the 3‐year survival AUC was 0.881 (Figure 3B), and the 5‐year survival AUC was 0.876 (Figure 3C). These findings suggest that EZH2 expression alone is sufficient to predict survival of glioma patients. Therefore, we constructed a nomogram integrating both EZH2 expression and clinicopathologic variables to provide a quantitative approach for predicting prognostic risk (Figure 3D). Using this nomogram, we predicted 1‐, 3‐ and 5‐year survival rates and assessed the predictive abilities with C‐index. As shown in Figure 3E‐G, the C‐index was 0.8007, indicating that our nomogram has a medium accuracy in survival predictability. Therefore, our results verify that the EZH2 expression level can be used as a prognostic indicator, and our nomogram can be used to evaluate prognosis by considering EZH2 in combination with other parameters.

### 3.4 Identification of EZH2‐related signalling pathways and genes

**FIGURE 4:**
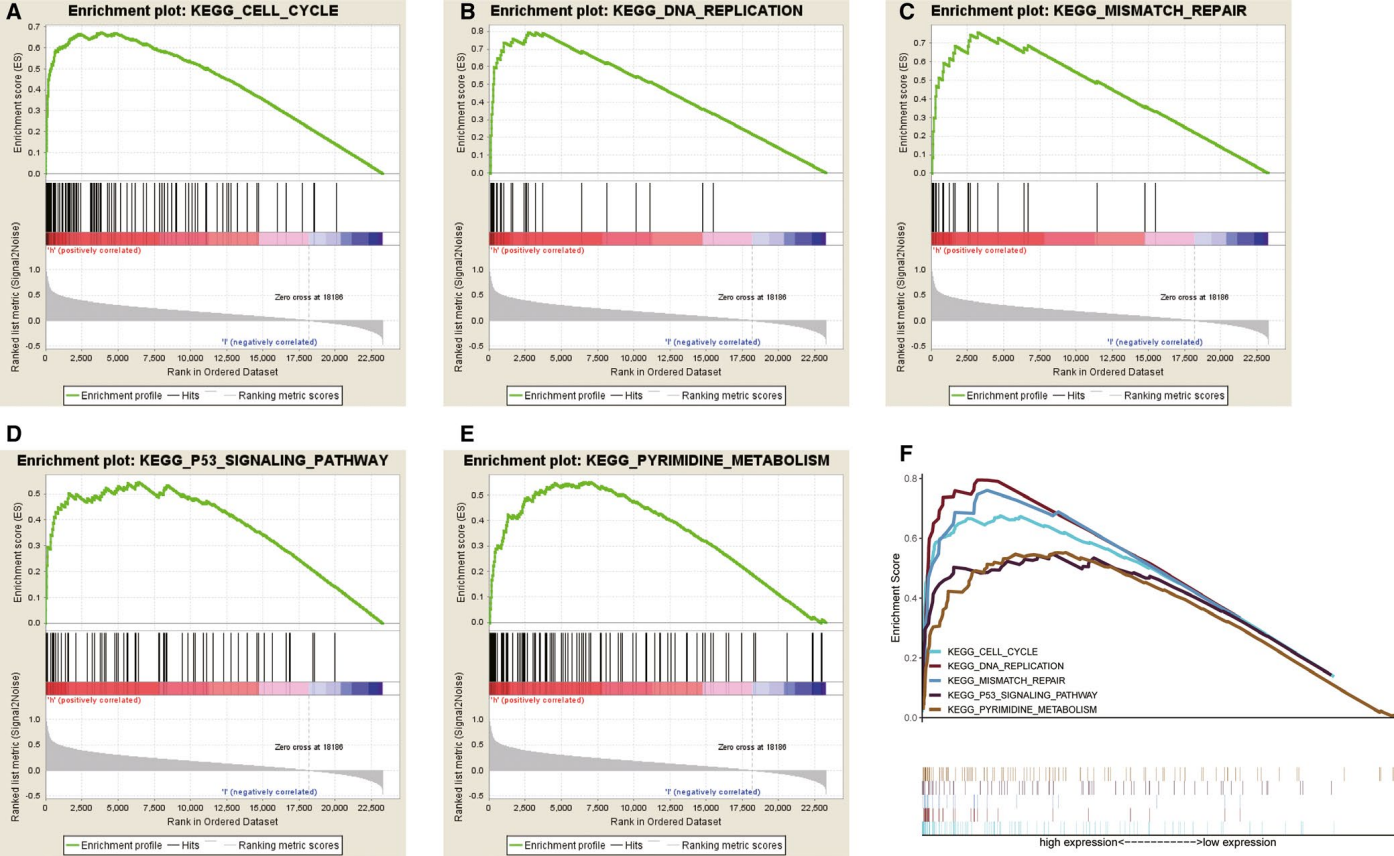
Enrichment of pathways and genes identified by gene set enrichment analysis (GSEA). (A‐E) The KEGGs cell cycle (A), DNA replication (B), mismatch repair (C), p53 signalling pathway (D) and pyrimidine metabolism (E) are differentially enriched in EZH2‐related glioma. (F) The five most highly enriched signalling pathways based on their NES. (ES, enrichment score; NES, normalized ES; and NOM *P*‐val, normalized *P*‐value)

To determine how EZH2 contributes to glioma pathogenesis, we performed Gene Set Enrichment Analysis (GSEA) of tissues with different EZH2 expression levels. Based on the normalized enrichment score (NES) and FDR q‐val (FDR <0.01), we selected the most significantly enriched biological pathways. High expression of EZH2 was associated with essential signalling pathways, including cell cycle, DNA replication, mismatch repair, p53 signalling and pyrimidine metabolism (Figure 4 and Table S2). EZH2 expression was confirmed to be positively associated with p53 gene expression (Pearson's *r* = 0.642, *P* < .001, Figure S2).

**FIGURE 5:**
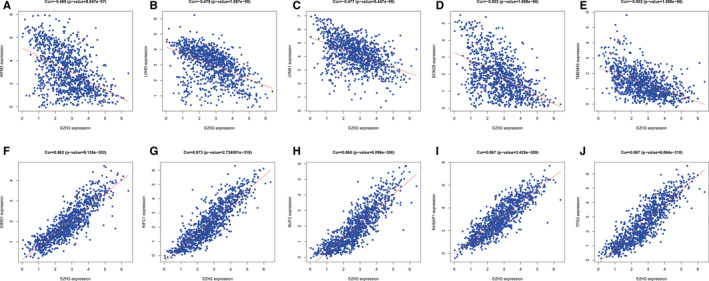
Correlation of EZH2 expression in glioma with the expression of other genes. (A‐J) The expression of EZH2 in glioma samples from the CGGA dataset showed positive correlations with GINS1 (A), KIFC1 (B), NUF2 (C), NUSAP1 (D) and TPX2 (E); and negative correlations with AIFM3 (F), LDHD (G), LYNX1 (H), SCN2B (I) and TMEM56 (J)

To further evaluate mechanisms associated with EZH2 expression, we examined EZH2‐related genes based on the CGGA data set. As shown in Figure 5A‐E, the five most negatively relevant genes were AIFM3 (Pearson's *r* = −0.469, *P* < .001), LDHD (Pearson's *r* = −0.479, *P* < .001), LYNX1 (Pearson's *r* = −0.477, *P* < .001), SCN2B (Pearson's *r* = −0.503, *P* < .001) and TMEM56 (Pearson's *r* = −0.503, *P* < .001). By contrast, the five most positively relevant genes were GINS1 (Pearson's *r* = 0.862, *P* < .001), KIFC1 (Pearson's *r* = 0.873, *P* < .001), NUF2 (Pearson's *r* = 0.865, *P* < .001), NUSAP1 (Pearson's *r* = 0.867, *P* < .001) and TPX2 (Pearson's *r* = 0.867, *P* < .001) (Figure 5F‐J). Further analysis of these ten genes shows that the five positively relevant genes were positively associated with each other and negatively associated with five negatively relevant genes (Figure S3). These results provide insight into potential underlying mechanisms of EZH2 in the pathogenesis of glioma.

### 3.5 Relationship of immune response and glioma

**FIGURE 6:**
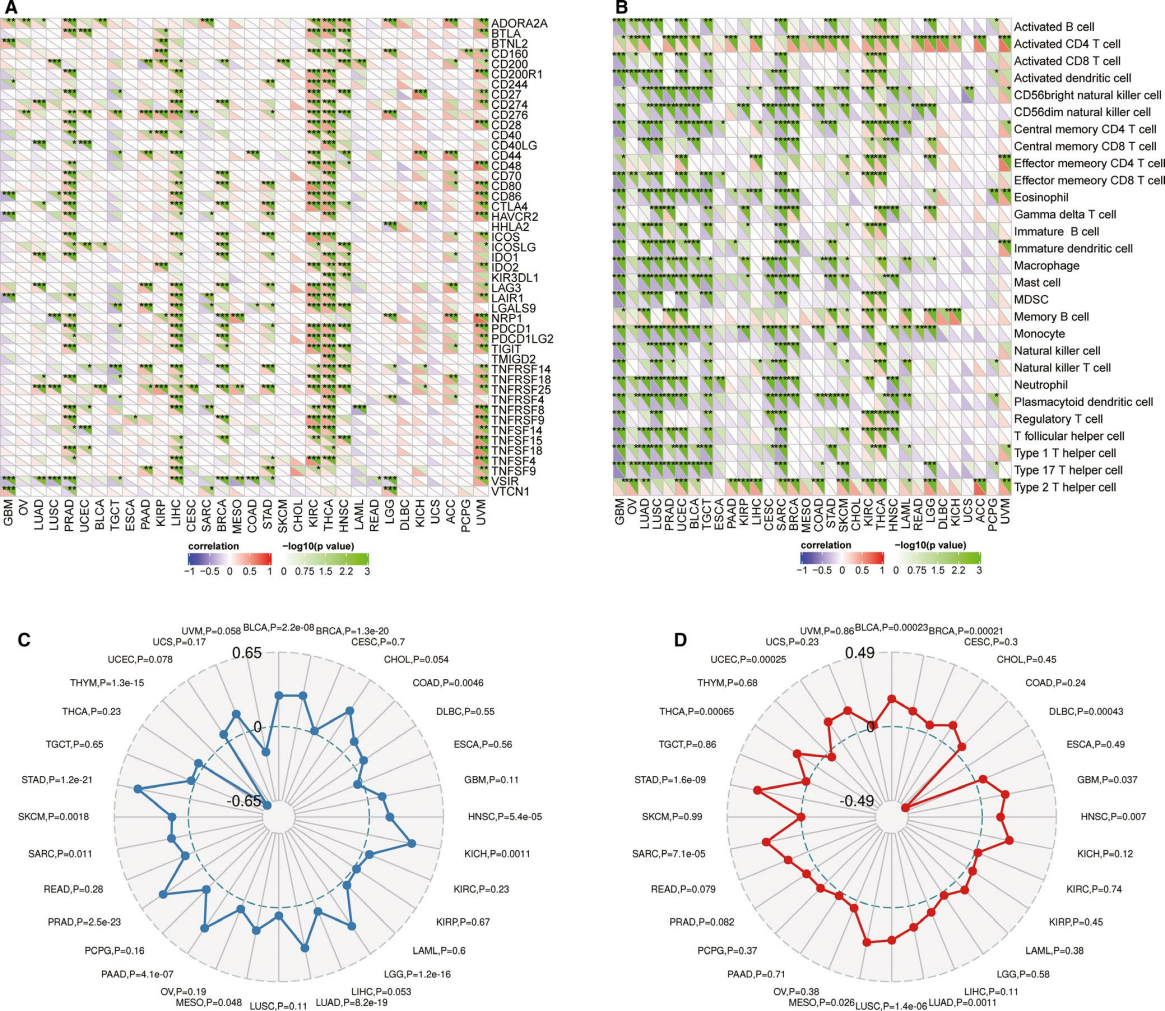
Immune relevance of EZH2 in glioma patients. (A) Expression of EZH2‐related immune checkpoint genes in different tumours; (B) Expression of EZH2‐related TIL marker genes in different tumours; (C) EZH2 had no relevance with the tumour mutation burden (TMB); (D) EZH2 expression is associated with microsatellite instability (MSI) in LGG but not GBM

Immunotherapy, including therapeutic vaccines and engineered T cells based on tumour‐immune cell interactions and checkpoint blockers, has been a focus of many recent strategies for treating glioma and other cancers.^7^, ^8^, ^9^, ^10^, ^11^ Therefore, we assessed the expression of immune checkpoint genes and marker genes from TILs. In LGG, ADORA2A, CD160, CD276, NRP1 and VTCN1 were among the immune‐related genes that were significantly overexpressed, while expression of HHLA2 and VSIR was decreased. However, in GBM, BTNL2 and VTCN1 expression was increased obviously, while the expression of CD86, HAVCR2, LAIR1 and VSIR was significantly decreased (Figure 6A). Furthermore, activated CD4 T cells, gamma delta T cells, memory B cells and type 2 T helper cells were positively correlated, and activated B cells, CD56dim natural killer cells, monocytes and type 17 T helper cells were negatively correlated in LGG, while in GBM, activated CD4 T cells and memory B cell type 2 T helper cells were also positively correlated, and activated B cells, activated CD8 T cells, activated dendritic cells, central memory CD4 T cells, effector memory CD8 T cells, eosinophils, immature B cells, immature dendritic cells, macrophages, mast cells, myeloid‐derived suppressor cells (MDSCs), monocytes, natural killer cells, natural killer T cells, neutrophils, plasmacytoid dendritic cells, regulatory T cells, T follicular helper cells, type 1 T helper cells and type 17 T helper cells were all negatively correlated (Figure 6B). These results verify that GBM has a more immunosuppressive profile than LGG.

It has been reported that TMB and MSI are independent prognostic factors for glioma and that TMB is associated with grade, age, subtype and mutations affecting genomic structure.^13^, ^26^ Therefore, we evaluated the correlation between TMB, MSI and EZH2. By Pearson correlation analysis, we found that TMB was not significantly correlated to EZH2 expression in either LGG (*P* = 1.2e‐16) or GBM (*P* = .11) (Figure 6C). Similarly, MSI was not associated with EZH2 expression in LGG (*P* = .58) or GBM (*P* = .037) (Figure 6D).

### 3.6 Association between EZH2 expression, immune infiltration and the tumour microenvironment

**FIGURE 7:**
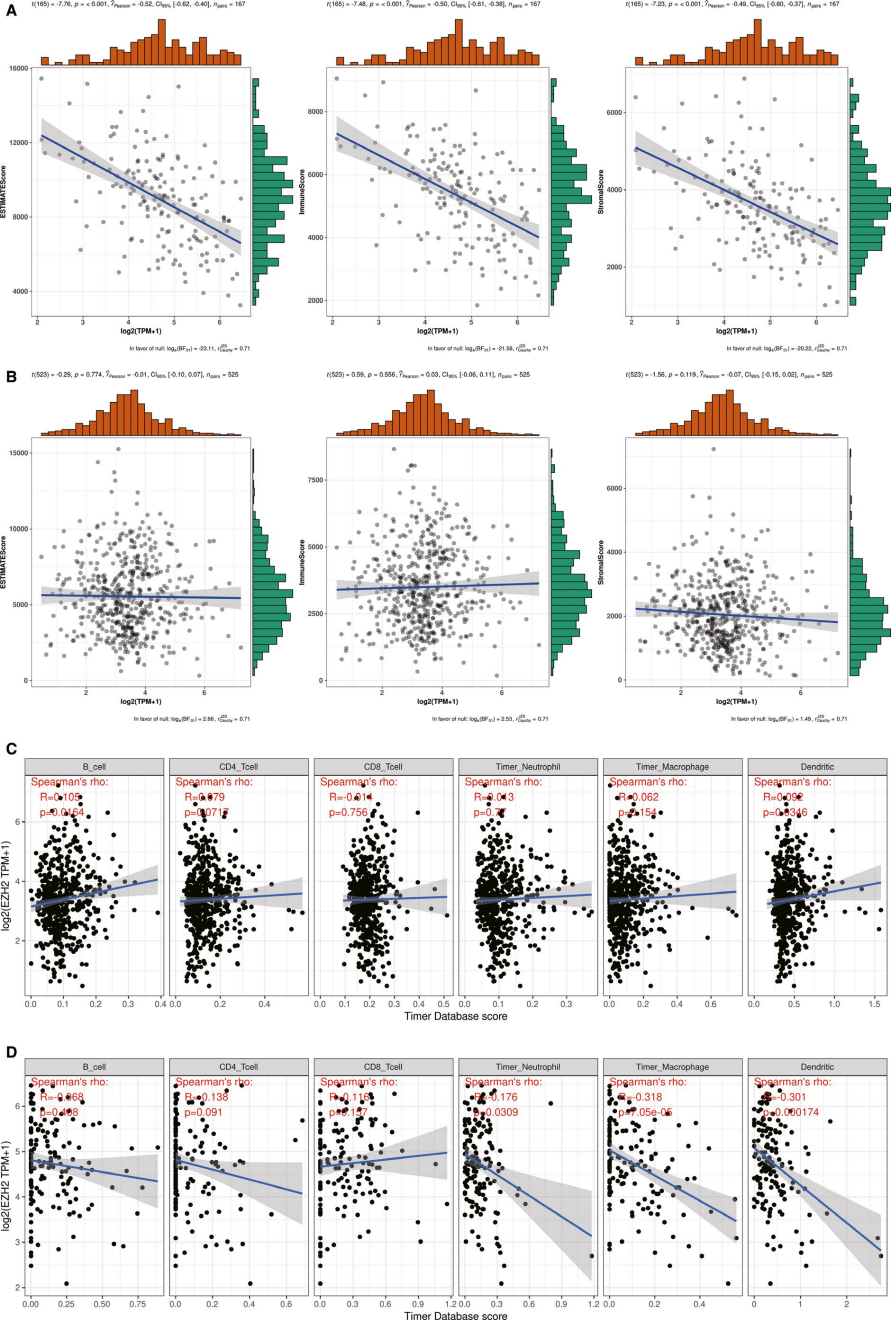
Association between EZH2 expression, immune infiltration and the tumour microenvironment. (A) and (B) Associations between EZH2 and the tumour microenvironment including immune cells, stromal cells, and both of them in GBM and LGG. (C) and (D) Associations between EZH2 and immune infiltration in LGG and GBM

To further determine potential mechanisms associated with EZH2 expression, we calculated the ESTIMATEscore, immune score and stromal score of LGG and GBM by using the ESTIMATE algorithm. The immune scores were statistically higher in GBM compared with LGG; and EZH2 was more relevant in GBM (*P *= −0.52) than LGG (*P *= −0.01) (Figure 7A‐B). As added confirmation, we detected infiltration of immune cells with the TIMER database (Figure 7C‐D). EZH2 expression was positively correlated with B cell and dendritic infiltration in LGG, while in GBM, EZH2 expression was more significantly correlated with macrophage and dendritic infiltration.

## 4 DISCUSSION

In this study, we verified that EZH2 is significantly up‐regulated in glioma tissues and cell lines.^23^ Furthermore, using a population of 681 glioma patients from the CGGA data, we verified that EZH2 expression is related to a poor overall survival^25^ and demonstrated the predictive ability of EZH2 expression by performing ROC analysis. To further understand the prognostic ability of EZH2 in glioma, we evaluated the association of EZH2 expression with clinicopathologic variables and established a nomogram based on multivariate Cox regression analysis of the 1‐, 3‐ and 5‐year survival of glioma patients. We also performed GSEA and immune profiling to elucidate potential mechanisms by which EZH2 promotes tumorigenesis. Our results support the utility of EZH2 expression as a potential predictor in the prognosis of glioma patients that functions through a variety of mechanisms to promote immune evasion and tumour progression.

As the most common and malignant primary intracranial tumour, glioma represents about 28% of all primary brain tumours and approximately 80% of malignant brain tumours.^1^ Although combined surgery with focal fractionated radiotherapy and the adjuvant temozolomide has been developed as the standard first line treatment, the 5‐year survival of GBM patients is only 5%.^3^ Therefore, it is urgent to explore new reliable prognostic targets for survival prediction and treatment of glioma. Our decision to evaluate the predictive ability of EZH2 was based on recent studies suggesting that it is an oncogene in glioma.^27^ Inhibition of EZH2 expression has been shown to strongly impair glioma cell self‐renewal in vitro and tumour‐initiating capacity in vivo, and to improve drug and radiotherapy resistance.^28^ However, the prognostic role of EZH2 in glioma had not been exactly demonstrated prior to this study.

Our results verify that EZH2 displays obviously higher expression in glioma tissues than in adjacent normal tissues. Furthermore, low EZH2 expression in glioma patients from the CGGA database was significantly related to better pathologic stage, histological grade and satisfactory survival time, which supports findings from other studies.^29^, ^30^, ^31^ Although the EZH2 expression level was correlated with other clinical parameters, our results demonstrate that EZH2 expression also may provide an independent predictor of overall survival in glioma patients. We constructed a risk score that could predict the prognosis of glioma patients by integrating the EZH2 expression with five clinicopathological variables including PRS type, Grade, Chemo status, IDH mutation and 1p19q status. We also confirmed the performance of the nomogram for predicting the 1‐, 3‐ and 5‐year survival, which had AUC’s of 0.853, 0.881 and 0.876, respectively.

To further explore how EZH2 is involved in glioma pathogenesis, we performed GSEA between tissues with different EZH2 expression levels and found that EZH2 expression was associated with essential signalling pathways, including cell cycle, DNA replication, mismatch repair, p53 signalling and pyrimidine metabolism. Additionally, we investigated the relationship of EZH2 and related genes based on the CGGA data set and determined that the five most negatively relevant genes were AIFM3, LDHD, LYNX1, SCN2B and TMEM56, while the five most positively relevant genes were GINS1, KIFC1, NUF2, NUSAP1 and TPX2. Importantly, previous studies reported that AIFM3, LDHD, LYNX1, SCN2B or TMEM56 were all negatively corelated with glioma,^32^, ^33^, ^34^, ^35^, ^36^ and GINS1, KIFC1, NUF2, NUSAP1 or TPX2 were both positively related to glioma progression.^37^, ^38^, ^39^, ^40^, ^41^ Previous studies have reported that G‐protein‐coupled receptor B1 (BAI1/ADGRB1) regulates the p53 signalling pathway in medulloblastoma.^42^ Furthermore, H3K27me3, a marker of the silent chromatin conformation, has been detected at the ADGRB1 promoter; and inhibition of EZH2, the catalytic component of the Polycomb Repressive complex 2 that methylates H3K27, switches the gene into an active chromatin status and reactivates BAI1 expression.^43^ Targeting of EZH2 promotes transition from H3K27me3 to H3K27ac at the promoter, recruits C/EBPβ (CREB‐binding protein; CBP) and CBP transcription factors, and activates ADGRB1 gene transcription.^43^ Therefore, further investigations need to be carried out to elucidate the mechanism of EZH2 in targeting the p53 signalling pathway to mediate glioma progression.

With specific anatomical and physiological features like the blood‐brain barrier, which facilitates selective entry of immune cells in the absence of lymphatic vessels, lymph nodes or critical immune organs in the periphery, the central nervous system was once considered immune‐privileged.^44^ Nevertheless, recent publications demonstrate the existence of lymphatic systems in the brain that communicate glioma antigens and immune cells between the brain and other immune components by alternate routes.^45^, ^46^, ^47^ Normally, tumour cells adopt different strategies to inhibit the immune system, so that they can ensure survival at various stages of anti‐tumour immune response.^48^ Consequently, tumour immunotherapy has been developed as a treatment modality that controls and eliminates tumours by restarting and maintaining the tumour immune cycle, restoring the normal tumour immune response, and controlling and eliminating tumours.^49^ Tumour immunotherapy may involve immuno‐checkpoint‐blocking monoclonal antibodies, cell therapy, DNA vaccines or small molecule inhibitors.^50^, ^51^, ^52^, ^53^ To evaluate the association of EZH2 expression within the glioma microenvironment, we evaluated the ImmuneScore, StromalScore or ESTIMATEScore. Our results suggest a moderate immune involvement in GBM but no relevance in LGG, indicating that the EZH2 phenotype involves immune suppression. We evaluated immune checkpoints that are known to be involved in glioma and calculated the association of these immuno‐checkpoints with EZH2. In both LGG and GBM, several immuno‐checkpoints correlated with EZH2 significantly, which provides novel potential targets for immune therapy. The TMB, defined as mutations per megabase, is a potential biomarker of immune checkpoint inhibitors in many cancer types, as neoantigens are generated by somatic tumour mutations.^54^ Therefore, we also analysed the relationship between EZH2 expression and TMB in glioma. Our results suggest that EZH2 expression was not significantly associated with TMB in LGG and GBM, with p values of 1.2e‐16 and 0.11, respectively.

There is also emerging evidence suggesting that TILs play important roles in the diagnosis and treatment of patients with glioma. TILs can promote or regulate tumour progression and growth by means of different types of cells that interact.^55^ In this study, we evaluated EZH2 expression‐based immune infiltration and found that most immune cells were correlated negatively with the expression of EZH2, especially in GBM. Furthermore, we downloaded the data sets of 6 immune‐infiltrating cells from the TIMER database and analysed the correlation with EZH2 expression. EZH2 expression was positively correlated with B cell and dendritic infiltration in LGG; however, EZH2 expression was correlated more significantly with macrophage and dendritic infiltration in GBM. These observations indicate that the microenvironment of glioma was in an immunosuppressive state, corroborating the significance of the immune system in glioma development.

Lastly, we evaluated correlations between EZH2 expression and MSI. Microsatellites are short, repeated DNA sequences that are extremely prone to DNA replication errors and are readily corrected by the MMR system.^56^ DNA MMR deficiency causes genomic instability and results in the accumulation of numerous mutations in microsatellite sequences that lead to MSI. Furthermore, MSI has been investigated as a molecular mechanism that is indicative of the prognosis and treatment response in glioma.^26^ Here, we used Spearman's correlation analysis to evaluate the relationship between EZH2 expression and glioma MSI; however, there was no relevance between EZH2 expression and MSI in GBM. On the contrary, EZH2 was related to MSI in LGG.

## 5 CONCLUSIONS

Overall, our results indicate that EZH2 may serve as a favourable prognostic factor for glioma patients. We also discovered that the p53 signalling pathway may be a primary pathway regulated by EZH2. Importantly, our nomogram showed a satisfactory predictive ability for EZH2 alone or in combination with other clinical parameters. Furthermore, the immune response was preliminarily indicated to underlie glioma progression, which suggests novel approaches in glioma immune therapy. Further prospective experiments should be carried out to explore the molecular and immune mechanisms of EZH2 in glioma.

## CONFLICT OF INTEREST

No conflict of interest exits in the submission of this manuscript.

## AUTHOR CONTRIBUTIONS

**Chen Yinan:** Conceptualization (lead); Data curation (equal); Formal analysis (equal); Funding acquisition (lead); Investigation (equal); Project administration (equal); Writing‐original draft (lead). **Hou Shiqiang:** Data curation (lead); Formal analysis (equal); Investigation (equal); Methodology (equal); Software (equal); Writing‐original draft (equal). **Jiang Rui:** Methodology (equal); Resources (supporting); Software (supporting). **Sun Junlong:** Data curation (supporting); Formal analysis (supporting); Investigation (supporting); Software (equal). **Cheng Chuandong:** Conceptualization (supporting); Project administration (supporting); Resources (equal); Supervision (equal); Validation (equal); Visualization (equal); Writing‐review & editing (supporting). **Qian Zhongrun:** Conceptualization (equal); Funding acquisition (equal); Project administration (equal); Supervision (equal); Writing‐review & editing (lead).

## CONSENT FOR PUBLICATION

This manuscript has been approved by all authors for publication.

## Supporting information

Fig S1

Fig S2

Fig S3

Tab S1

Tab S2

## Data Availability

Additional Supporting Information may be found online in the supporting information tab for this article.
