# Supplementary figures and images for: EZH2 is a potential prognostic predictor of glioma
Source: J Cell Mol Med. 2020 Dec 4;25(2):925–36. doi: 10.1111/jcmm.16149 (PMC7812280; doi:10.1111/jcmm.16149)

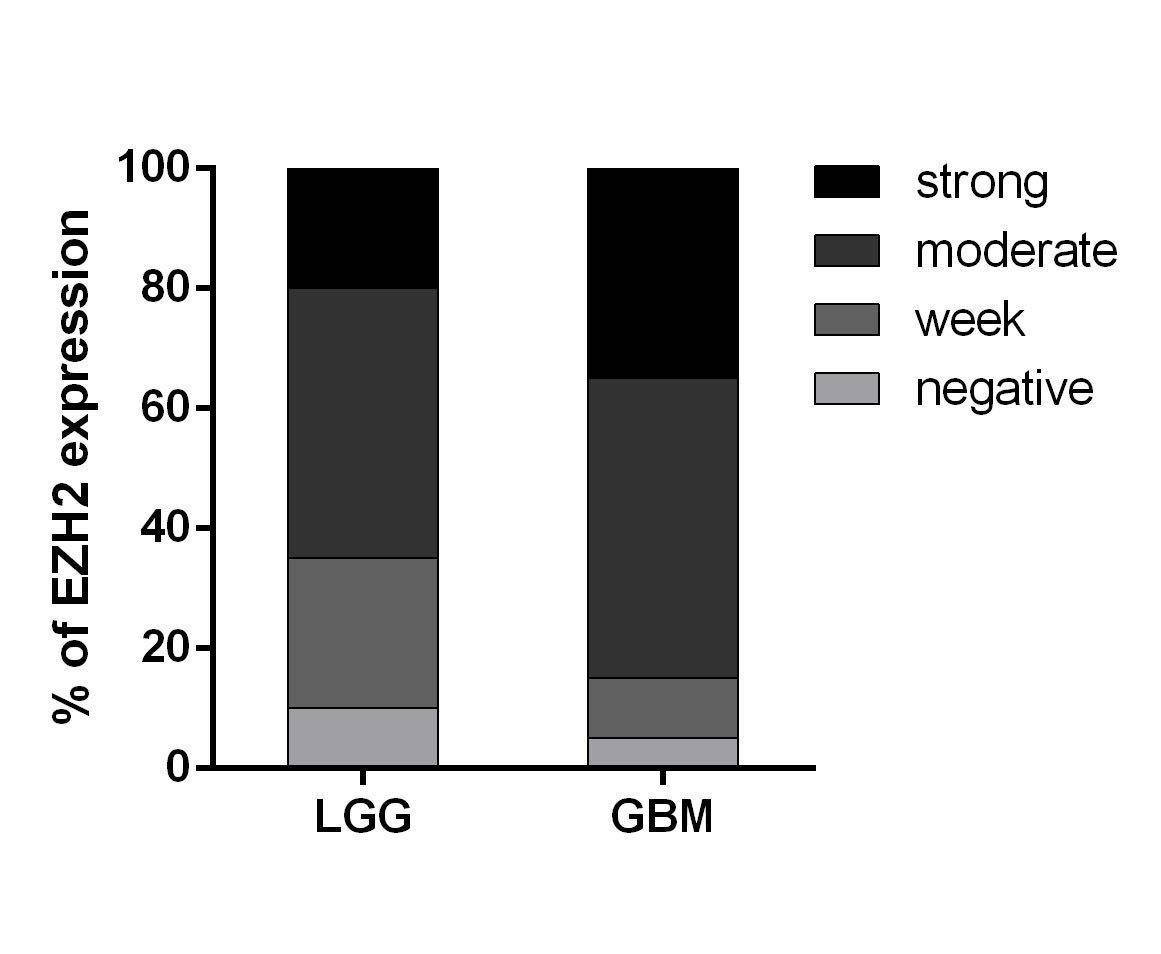

Supplement: Supplementary file 1 — Fig S1 [file JCMM-25-925-s001.tif]

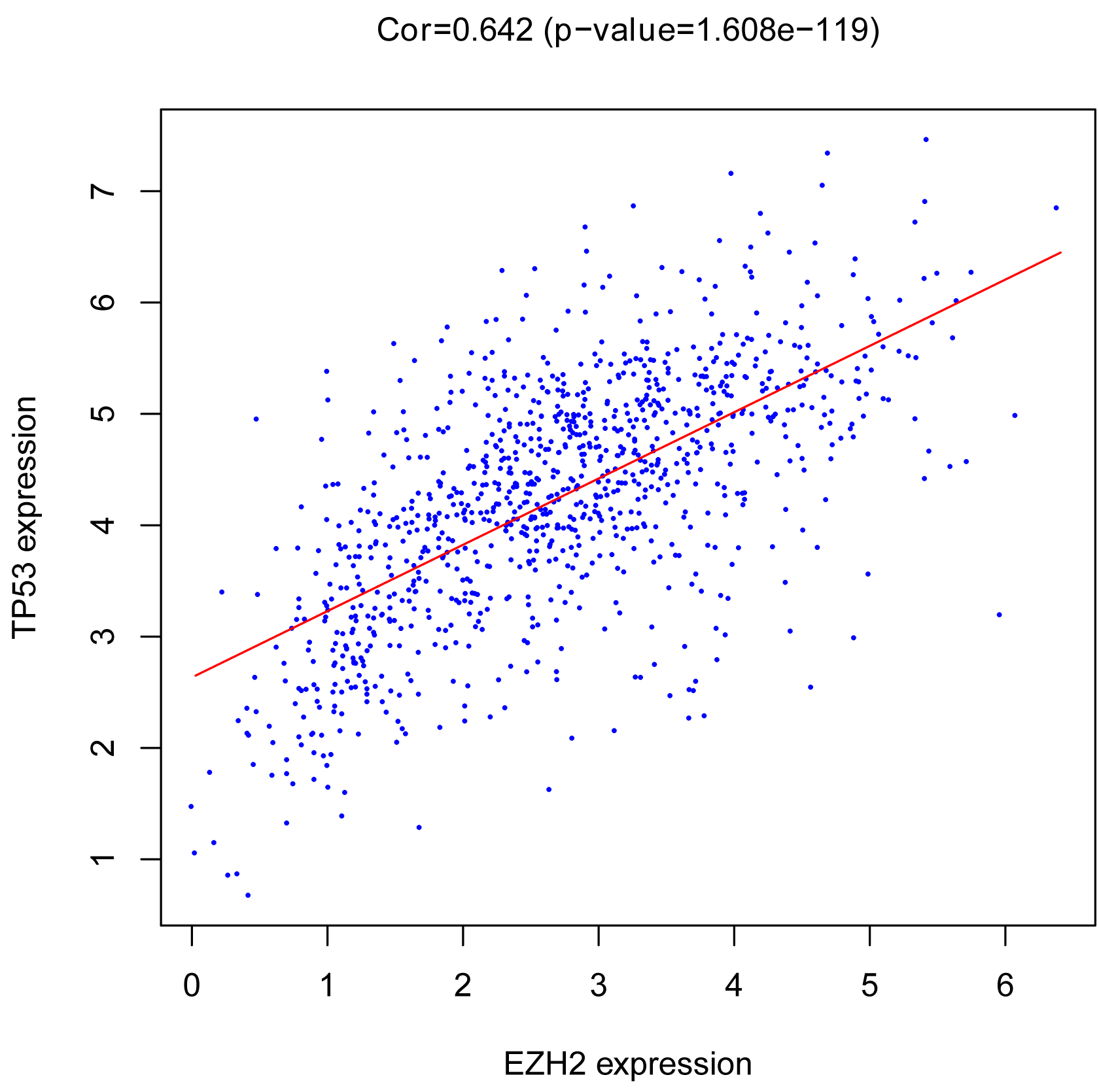

Supplement: Supplementary file 2 — Fig S2 [file JCMM-25-925-s002.tif]

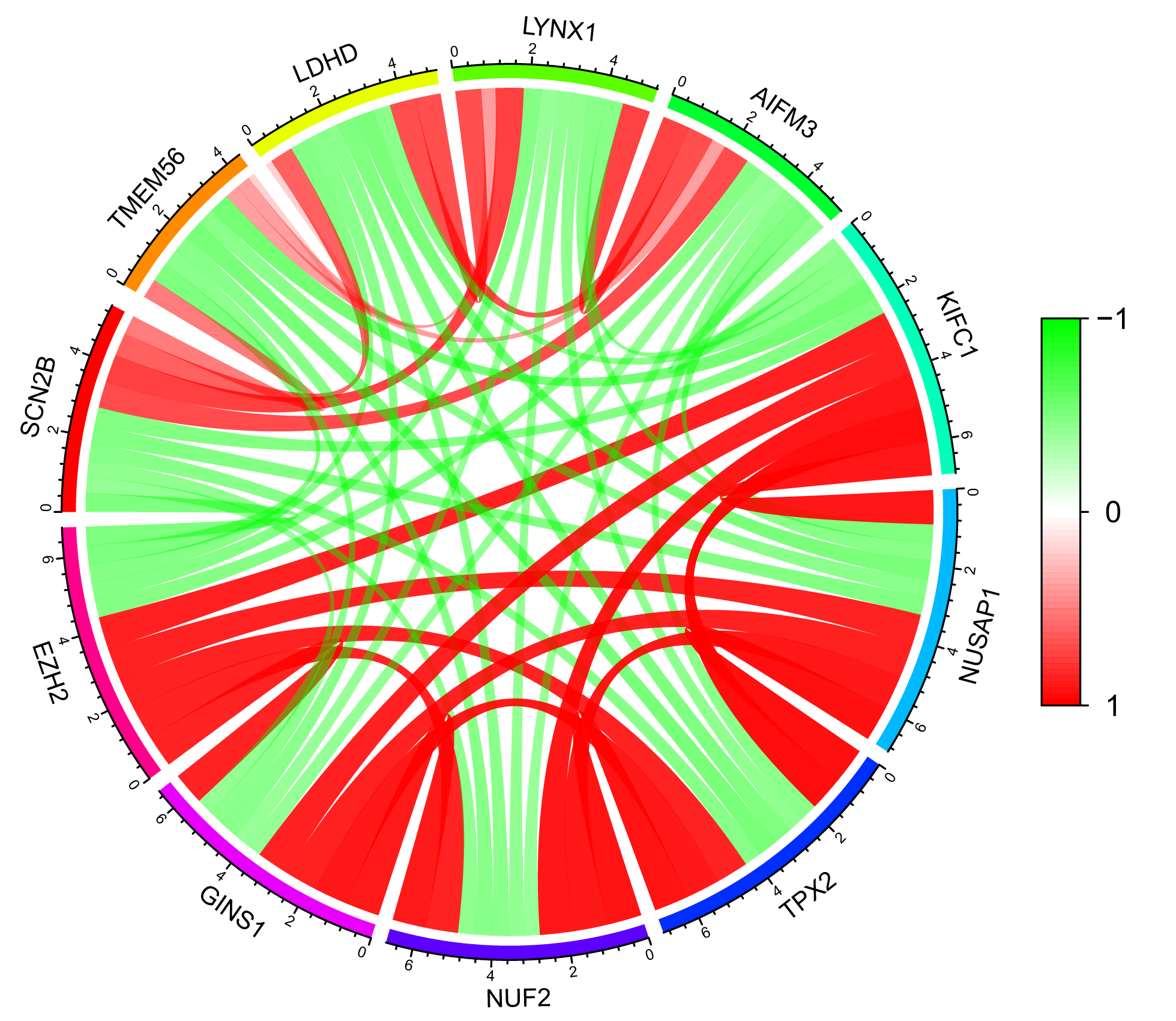

Supplement: Supplementary file 3 — Fig S3 [file JCMM-25-925-s003.tif]
