# Supplementary material for: EZH2 is a potential prognostic predictor of glioma
Source: J Cell Mol Med. 2020 Dec 4;25(2):925–36. doi: 10.1111/jcmm.16149 (PMC7812280; doi:10.1111/jcmm.16149)
Supplement: Supplementary file 4 — Tab S1 [file JCMM-25-925-s004.docx]

| **Variable** | **Univariate analysis** | | | **Multivariate analysis** | | |
| --- | --- | --- | --- | --- | --- | --- |
|  | **HR** | 95% CI | ***p*** | HR | 95% CI | ***p*** |
| **PRS type** | 2.123 | 1.818-2.478 | **0.000** | 1.973 | 1.678-2.320 | **0.000** |
| **Histology** | 4.487 | 3.695-5.449 | **0.000** | 0.696 | 0.447-1.083 | 0.108 |
| **Grade** | 2.883 | 2.526-3.291 | **0.000** | 2.443 | 1.776-3.360 | **0.000** |
| **Gender** | 1.044 | 0.866-1.258 | 0.655 | 1.056 | 0.873-1.227 | 0.575 |
| **Age** | 1.624 | 1.345-1.960 | **0.000** | 1.247 | 1.022-1.521 | 0.029 |
| **Radio status** | 0.929 | 0.720-1.199 | 0.571 | 0.909 | 0.694-1.190 | 0.488 |
| **Chemo status** | 1.647 | 1.328-2.044 | **0.000** | 0.659 | 0.517-0.839 | **0.001** |
| **IDH mutation** | 3.153 | 2.606-3.816 | **0.000** | 1.839 | 1.456-2.323 | **0.000** |
| **1p19q status** | 4.337 | 3.179-5.917 | **0.000** | 2.410 | 1.725-3.367 | **0.000** |
| **EZH2** | 1.552 | 1.426-1.688 | **0.000** | 1.228 | 1.125-1.340 | **0.000** |

**Table S1** Based on univariate Cox and multivariate Cox regression analysis, EZH2 expression was correlated with overall survival as an independent factor.
