# Supplementary material for: EZH2 is a potential prognostic predictor of glioma
Source: J Cell Mol Med. 2020 Dec 4;25(2):925–36. doi: 10.1111/jcmm.16149 (PMC7812280; doi:10.1111/jcmm.16149)
Supplement: Supplementary file 5 — Tab S2 [file JCMM-25-925-s005.docx]

| Gene set name | NES | NOM p-val | FDR q-val |
| --- | --- | --- | --- |
| KEGG_CELL_CYCLE | 2.010 | 0.000 | 0.037 |
| KEGG_DNA_REPLICATION | 1.944 | 0.000 | 0.044 |
| KEGG_MISMATCH_REPAIR | 1.895 | 0.000 | 0.037 |
| KEGG_P53_SIGNALING_PATHWAY | 1.684 | 0.010 | 0.148 |
| KEGG_PYRIMIDINE_METABOLISM | 1.745 | 0.002 | 0.141 |

**Table S2** High expression of EZH2 was associated with essential signaling pathways, including cell cycle, DNA replication, mismatch repair, p53 signaling and pyrimidine metabolism.
